# Geographic variability in contemporary utilization of PET imaging for prostate cancer: a medicare claims cohort study

**DOI:** 10.1186/s40644-025-00898-6

**Published:** 2025-07-04

**Authors:** Stephan M. Korn, Zhiyu Qian, Hanna Zurl, Nathaniel Hansen, Klara K. Pohl, Daniel Stelzl, Filippo Dagnino, Stuart Lipsitz, Jianyi Zhang, Adam S. Kibel, Caroline M. Moore, Kerry L. Kilbridge, Shahrokh F. Shariat, Stacy Loeb, Hebert Alberto Vargas, Quoc-Dien Trinh, Alexander P. Cole

**Affiliations:** 1 Department of Urology, Medical University of Vienna, Vienna, Austria; 2 Center for Surgery and Public Health, Brigham and Women’s Hospital, Harvard Medical School, Boston, MA USA; 3 Department of Urology, Mass General Brigham, Harvard Medical School, Boston, MA USA; 4 Department of Urology, Medical University of Graz, Graz, Austria; 5 Department of Urology, University Medical Center Hamburg-Eppendorf, Hamburg, Germany; 6 Department of Urology, Humanitas Clinical and Research Hospital, Milan, Italy; 7 University College London, London, UK; 8 Lank Center for Genitourinary Oncology, Dana-Farber Cancer Institute, Boston, MA USA; 9 Karl Landsteiner Institute of Urology and Andrology, Vienna, Austria; 10 Department of Urology, Comprehensive Cancer Center, Medical University of Vienna, Vienna, Austria; 11 Department of Urology and Population Health, New York University Langone Health and Manhattan Veterans Affairs, New York, NY USA; 12 Department of Radiology, New York University Langone Health, New York, NY USA; 13 Department of Urology, University of Pittsburgh, Pittsburgh, USA

**Keywords:** Prostate cancer, Rural population, Urban population, Positron-emission tomography, Healthcare disparities

## Abstract

**Background:**

Potential rural-urban differences in prostate cancer care are understudied, particularly regarding the utilization of advanced diagnostic tests. Herein we examined variations in Positron Emission Tomography (PET) utilization for prostate cancer care, including diagnosis, staging and treatment planning, across residential regions in the United States.

**Methods:**

Patients newly diagnosed with prostate cancer between 2019 and 2021 and post-diagnostic PETs were identified using full Medicare claims data. PET use was assessed in all newly diagnosed patients, though indications vary by risk. Patients’ counties were categorized as metro, urban, or rural, from most to least urbanized. Regional PET utilization was further examined at the level of hospital referral regions. A multivariable logistic regression model was performed to assess the impact of rurality on PET imaging. A secondary analysis included an interaction term for race to explore the effect of residence on PET imaging by racial group.

**Results:**

Overall, 495 865 patients were included in the analysis: 393 861 (79.4%) lived in metro, 56 698 (11.4%) in urban and 39 707 (8.0%) in rural counties. Patients in metro counties underwent PET imaging more often (8.4%) than patients in urban (7.3%) or rural counties (7.2%), *p* < 0.0001. At a level of hospital referral region, PET utilization rates ranged from 2.2 to 20.8%. PET imaging was more commonly performed in White compared to Black or Hispanic patients. Rural patients were less likely to undergo PET imaging compared to metro patients (odds ratio [OR] 0.87, 95% Confidence interval [CI]: 0.82–0.92 *p* < 0.0001). Rural Black (OR 0.69, 95%CI 0.57–0.83, *p* < 0.0001) and rural White patients (OR 0.89, 95%CI 0.83–0.94 *p* < 0.0001) were less likely to obtain PET imaging compared to their metro counterparts, p-interaction < 0.0001.

**Conclusion:**

Rural patients were less likely to undergo PET imaging than metro patients. The effect of rurality was most pronounced among Black patients. Our findings underscore the need for strategies to support equitable use of PET imaging.

**Supplementary Information:**

The online version contains supplementary material available at 10.1186/s40644-025-00898-6.

## Introduction

While racial disparities are well documented throughout prostate cancer’s disease course [1], geographic differences in access to care are less studied. However, rural patients with prostate cancer have been shown to have less guideline concordant diagnostic and therapeutic strategies [2]. Further, the interplay of racial and geographic disparities appears in prostate cancer [3]. Although survival has improved for patients with prostate cancer overall, rural non-Hispanic Black patients benefited more slowly and inconsistently than their urban and non-Hispanic White counterparts [4]. Disparities in prostate cancer care occur early and persist throughout the disease course [1]. Understanding differences in access to initial diagnostics is therefore crucial.

In recent years, there have been significant advances in the diagnosis and treatment of prostate cancer, in which imaging has played an important role [5]. In particular, the use of functional Positron Emission Tomography (PET) has gained increasing interest [6, 7]. Due to their higher accuracy in detecting metastases compared to standard imaging, PET scans are increasingly used for primary staging [8] and biochemical recurrence [9]. Moreover, PET-based radioligand therapy is a new treatment option in metastatic disease [10]. This highlights the growing importance of advanced imaging in both prostate cancer diagnosis and treatment [11]. The impact of geography on PET scan utilization is not well understood. PET imaging resources vary across the United States [12, 13]. However, the impact of these differences on PET scan utilization among patients in metro, urban, and rural areas is unclear. To date, no national analysis has examined residence-based differences in PET imaging for prostate cancer.

In this context, we sought to assess whether the use of PET imaging for newly diagnosed prostate cancer differed according to metro, urban and rural residence. Because performing a PET scan is capital-intensive and requires scanners and radiotracers, which might not be readily available in low volume rural hospitals, we hypothesized that there would be significantly lower utilization of PET scans among rural patients. This study aims to fill the gap in understanding potential disparities in access to advanced prostate cancer imaging. In a secondary analysis, we also aimed to examine racial disparities in the context of residential setting.

## Methods

### Data source

We retrospectively identified patients using 100% Medicare claims data from the Research Identifiable Files, including the Beneficiary Summary File, accessed via the Centers for Medicare and Medicaid Services Virtual Research Data Center. This dataset includes patients’ demographics as well as detailed, longitudinal patient-level claims across inpatient, outpatient, and physician services covered by Medicare, the federal health insurance program primarily serving individuals aged 65 and older. It allows for comprehensive analysis of diagnostic and treatment patterns, including our primary outcome.

For regional analysis, we used patient-level Dartmouth Atlas hospital referral regions (HRR) [14]. Each HRR encompasses a hospital service area regardless of rural or urban setting and contains tertiary hospitals [15].

### Study cohort

The study cohort encompassed patients with incident prostate cancer diagnosed during the 2019–2021 period, identified through International Classification of Diseases, 10th revision (ICD-10) Clinical Modification topography classification C61. Among the identified patients, we reviewed 2018 records to exclude individuals with pre-existing prostate cancer diagnoses, beneficiaries younger than 66 years, and those with insufficient Medicare coverage duration (< 12 months). This approach ensured that our cohort included only incident prostate cancer cases, irrespective of other preexisting conditions. Each patient was counted only once, based on the year of their first prostate cancer diagnosis. We further excluded patients with missing ZIP code information. The final cohort comprised of all patients newly diagnosed with prostate cancer between January 1, 2019, and December 31, 2021. Supplementary Fig. 1 outlines the cohort selection, including eligibility and exclusion criteria.

### Dependent variable

To capture all PET scans performed for newly diagnosed prostate cancer, regardless of stage or risk group, the dependent variable was defined as receipt of post-diagnostic PET imaging during the study period, with follow-up limited accordingly to a maximum of 36 months. PET imaging included C11-Choline-, 18 F-fluciclovine-, 68Ga-prostate-specific membrane antigen (PSMA)-11-, 18 F-Piflufolastat-, and 18 F-fluorodeoxyglucose- (FDG-) PET Computed Tomography and/or PET Magnetic resonance imaging (MRI). PET utilization was identified based on global claims using Current Procedural Terminology (CPT) and Healthcare Common Procedure Coding System (HCPCS) codes (Supplementary Table 1). To avoid duplicate entries from separately billed technical and professional components or multiple related codes for a single imaging event, PET use was recorded as a binary indicator (yes/no) per patient.

### Predictor variable

Patient residence counties were classified as metro, urban, or rural based on Federal Information Processing System (FIPS) codes and the 2023 Rural-Urban Continuum Codes (RUCC) developed by the United States Department of Agriculture [16]. This classification incorporates population size, commuting patterns, and economic integration based on Office of Management and Budget definitions and differentiates between metropolitan and non-metropolitan counties. In our study, counties were categorized as follows: “metro” encompassing large urban areas (metropolitan RUCC codes 1–3), “urban” including intermediate-sized communities or suburban areas (non-metropolitan RUCC codes 4–6), and “rural”, representing the least urbanized regions with smaller or dispersed populations (non-metropolitan RUCC codes 7–9). This categorization follows established methods and allows for a more granular assessment of geographic disparities in care [17–22].

### Covariates

Our analysis included sociodemographic and geographic covariates such as race/ethnicity (Medicare race code), age, diagnosis year, and dual Medicare/Medicaid eligibility criteria, categorized as in Table 1. We characterized patients’ baseline health status by quantifying their pre-existing comorbidities as outlined by Medicare’s tracked chronic conditions. The conditions represent both physical (cardiovascular, respiratory, musculoskeletal) and mental health diagnoses, as well as neurological disorders and cancer. Each patient’s disease burden was systematically classified based on the number of conditions (0, 1, 2, or ≥ 3 concurrent conditions, excluding prostate cancer). Patients’ ZIP codes were matched to HRRs using Dartmouth Atlas data [14].

### Statistical analysis

We compared baseline covariates between patients in metro, urban and rural counties and in patients with and without PET imaging. For each categorical variable, we reported proportions. The distribution of categorical covariates between patients with and without PET imaging was compared using the chi-square test. A temporal validation analysis using histogram visualization was conducted to compare with baseline PET imaging, supporting the inference that PET scans were performed as part of diagnostic staging for prostate cancer. For our dependent variable, obtaining a PET scan, we then developed a patient-level multivariable logistic regression model. We specified HRR as the clustering unit in the model. The standard errors, 95% confidence intervals, and *p*-values accounted for clustering using generalized estimating equations, which addresses the correlation of outcomes from patients within the same HRR [23]. To visualize geographic variation in PET imaging, we generated a heatmap based on HRRs. PET actual utilization rates were calculated for each HRR among patients diagnosed with prostate cancer. HRRs were then grouped into percentile-based categories according to the national distribution of testing rates, allowing for comparison across regions with relatively low, average, or high utilization.

We included a residence-race interaction term in our model. This allowed us to assess whether the effect of residential setting on PET utilization varied between different racial groups. We analyzed the probability of obtaining a PET scan across different subgroups using a least squares mean adjusted probability analysis, which accounted for covariates and was clustered by HRR.

All statistical tests used an alpha level of 0.01 to determine significance, accounting for the large sample size and the potential for overpowered analyses that could lead to overinterpretation of modest associations.

All statistical analyses were conducted using SAS Enterprise Guide 7.1, and heatmaps were generated using SAS 9.4 (SAS Institute Inc., Cary, NC, USA).

## Results

We identified 495 865 patients newly diagnosed with prostate cancer from 2019 to 2021. Most patients lived in metro counties (*n* = 393 861 [79.4%]), while 56 698 (11.4%) patients lived in urban and 39 707 (8.0%) patients lived in rural counties. The average median age was 75 years (Interquartile range [IQR] 71–81). Most patients were White (*n* = 416 276 [84.0%]) and had ≥ 3 comorbidities (*n* = 403 183 [81.3%]). Prostate cancer diagnoses were highest in 2019 (*n* = 212 465 [42.9%]) vs. 2020 (*n* = 144 511 [29.1%]) and 2021 (*n* = 138 889 [28.0%]). Table 1 summarizes baseline characteristics by residential area.

**Table 1:** Patient baseline characteristics by residence

	Overall, *n*(%) *n* = 495 865 (100%)	Metro, *n*(%) *n* = 393 861 (79.4%)*	Urban, *n*(%) *n* = 56 698 (11.4%)*	Rural, *n*(%) *n* = 39 707 (8.0%)*	Unknown, *n*(%) *n* = 5 599 (1.1%)*
**Demographics and Health Status**					
**PET Imaging**					
Yes No	40 293 (8.1%) 455 572 (91.9%)	32 870 (8.4%) 360 991 (91.7%)	4 147 (7.3%) 52 551 (92.7%)	2 868 (7.2%) 36 839 (92.8%)	408 (7.3%) 5 191 (92.7%)
**Age Group**					
66–70 71–75 76–80 ≥ 81	122 665 (24.7%) 138 168 (27.9%) 104 083 (21.0%) 130 949 (26.4%)	96 932 (24.6%) 110 267 (28.0%) 82 632 (21.0%) 104 030 (26.4%)	14 426 (25.4%) 15 737 (27.8%) 11 779 (20.8%) 14 756 (26.0%)	10 078 (25.4%) 10 759 (27.1%) 8 492 (21.4%) 10 378 (26.1%)	1 229 (22.0%) 1 405 (25.1%) 1 180 (21.1%) 1 785 (31.9%)
**Race**					
White Black Hispanic Asian Other Unknown	416 276 (84.0%) 45 118 (9.1%) 4 930 (1.0%) 5 612 (1.1%) 9 762 (2.0%) 14 167 (2.9%)	325 251 (82.6%) 38 921 (9.9%) 4 459 (1.1%) 5 420 (1.4%) 8 014 (2.0%) 11 796 (3.0%)	50 451 (89.0%) 3 625 (6.4%) 280 (0.5%) 111 (0.2%) 929 (1.6%) 1 302 (2.3%)	35 748 (90.0%) 2 248 (5.7%) 139 (0.4%) 28 (0.1%) 733 (1.9%) 811 (2.0%)	4 826 (86.2%) 324 (5.8%) 52 (0.9%) 53 (1.0%) 86 (1.5%) 258 (4.6%)
**US Region**					
South West Midwest Northeast	180 800 (36.5%) 102 364 (20.6%) 104 985 (21.2%) 107 716 (21.7%)	142 700 (36.2%) 85 638 (21.7%) 74 184 (18.8%) 91 339 (23.2%)	21 882 (38.6%) 10 593 (18.7%) 16 242 (28.7%) 7 981 (14.1%)	16 111 (40.6%) 6 048 (5.2%) 14 500 (36.5%) 3 048 (7.7%)	107 (1.9%) 85 (1.5%) 59 (1.1%) 5 348 (95.5%)
**Dual eligibility for Medicaid**					
Yes No	34 267 (6.9%) 461 598 (93.1%)	26 750 (6.8%) 367 111 (93.2%)	3 684 (6.5%) 53 014 (93.5%)	3 179 (8.0%) 36 528 (92.0%)	654 (11.7%) 4 945 (88.3%)
**Disability as the original reason for Medicare entitlement**					
Yes No	43 310 (8.7%) 452 555 (91.3%)	31 927 (8.1%) 361 934 (91.9%)	6 134 (10.8%) 50 564 (89.2%)	4 824 (12.2%) 34 883 (87.9%)	425 (7.6%) 5 174 (92.4%)
**Year of Prostate Cancer Diagnosis**					
2019 2020 2021	212 465 (42.9%) 144 511 (29.1%) 138 889 (28.0%)	168 602 (42.8%) 114 635 (29.1%) 110 624 (28.1%)	24 431 (43.1%) 16 548 (29.2%) 15 719 (27.7%)	16 953 (42.7%) 11 768 (29.6%) 10 986 (27.7%)	2 479 (44.3%) 1 560 (27.9%) 1 560 (27.9%)
**Number of Chronic Conditions**					
0 1 2 ≥ 3	21 291 (4.3%) 26 949 (5.4%) 44 442 (9.0%) 403 183 (81.3%)	16 110 (4.1%) 20 575 (5.2%) 34 440 (8.7%) 322 736 (81.9%)	2 724 (4.8%) 3 435 (6.1%) 5 475 (9.7%) 45 064 (79.5%)	2 264 (5.7%) 2 657 (6.7%) 4 023 (10.1%) 30 763 (77.5%)	193 (3.5%) 282 (5.0%) 504 (9.0%) 4 620 (82.5%)

Baseline characteristics of patients in the overall cohort and by metro, rural, urban, and unknown counties of residence.; Percentages are given in column percentages unless indicated by * for row percentages, Abbreviations: PET = Positron Emission Tomograph

**Fig. 1:**
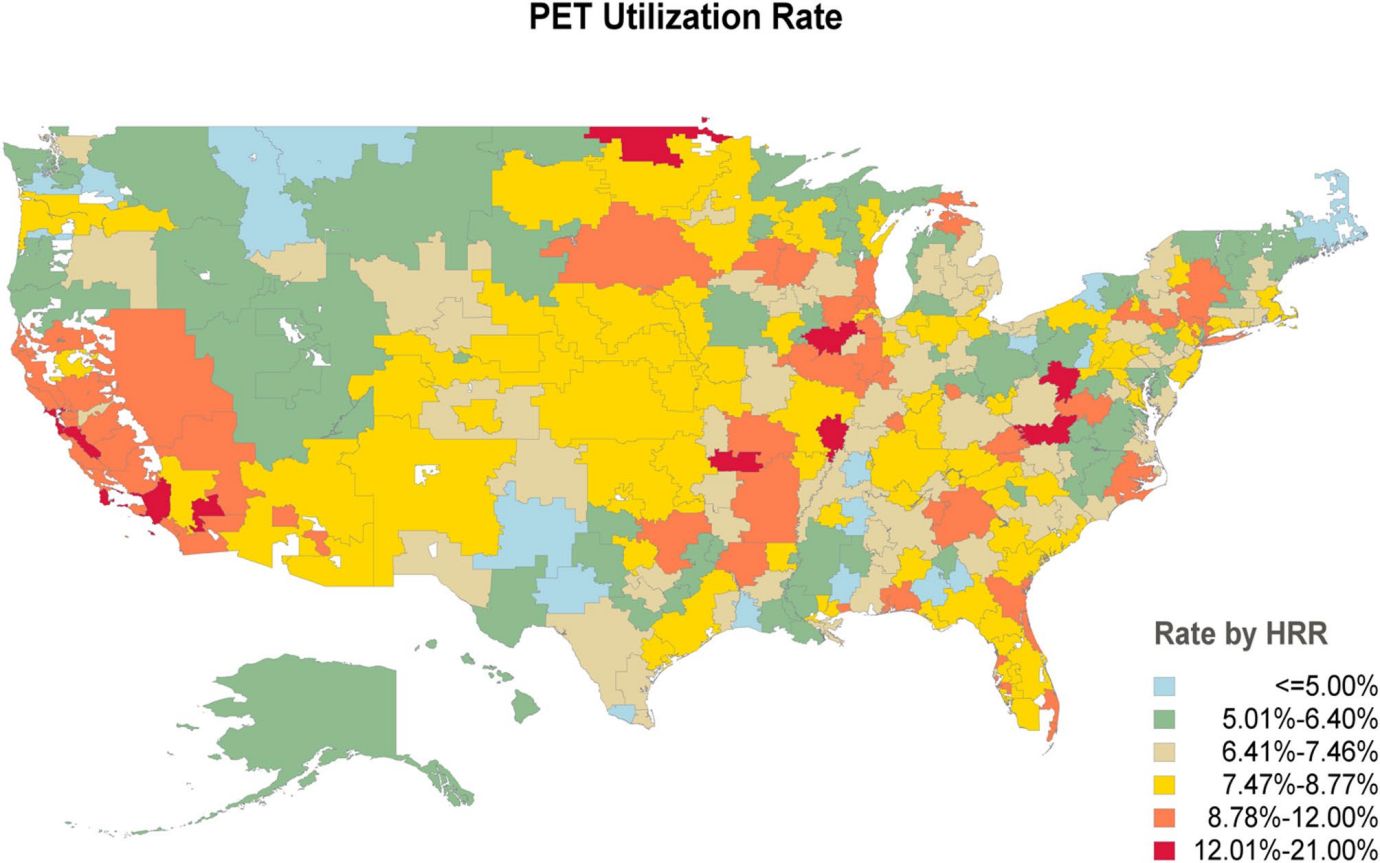
Heatmap based on hospital referral regions showing the rates of Positron Emission Tomography (PET) in patients with prostate cancer diagnosed 2019–2021. The data were categorized into 6 groups based on testing rate percentiles across the entire cohort: <5th percentile (corresponding to ≤ 5.00% germline genetic testing rate), 5th-25th percentile (5.01-6.40% testing rate), 25th-50th percentile (6.41-7.46% testing rate), 50th-75th percentile (7.47-8.77% testing rate), 75th-95th percentile (8.78–12.00% testing rate), and > 95th percentile (12.01-21.0% testing rate)

Overall, 40 293 (8.1%) patients underwent a PET scan during the study period. Across all 306 HRRs analyzed, PET utilization rates ranged from 2.2 to 20.8% (median 7.4%, IQR 6.4–8.7%). Figure 1 outlines PET utilization rate in individual HRRs.

Metro county patients obtained PET scans more frequently compared to those in urban or rural counties (*n* = 32 870 [8.4%] vs. *n* = 4 147 [7.3%] vs. *n* = 2 868 [7.2%], respectively, *p* < 0.0001). PET scans were relatively more frequent among White patients (*n* = 34 453 [8.3%]) compared to Black patients (*n* = 3 077 [6.8%]) and compared to Hispanic patients (*n* = 357 [7.2%]), *p* < 0.0001. Table 2 shows demographic parameters of patients with vs. without PET scan.

**Table 2:** Baseline characteristics of patients with and without PET imaging

	Overall, *n*(%) *n* = 495 865 (100%)	PET no, *n*(%) *n* = 455 572 (91.9%)	PET yes, *n*(%) *n* = 40 293 (8.1%)	*p*-value
**Demographics and Health Status**				
**Residence**				
Metro Urban Rural Unknown	393 861 (100%) 56 698 (100%) 39 707 (100%) 5 599 (100%)	360 991 (91.7%) 52 551 (92.7%) 36 839 (92.8%) 5 191 (92.7%)	32 870 (8.4%) 4 147 (7.3%) 2 868 (7.2%) 408 (7.3%)	< 0.0001
**Age Group**				
66–70 71–75 76–80 ≥ 81	122 665 (100%) 138 168 (100%) 104 083 (100%) 130 949 (100%)	113 094 (92.2%) 126 526 (91.6%) 94 613 (90.9%) 121 339 (92.7%)	9 571 (7.8%) 11 642 (8.4%) 9 470 (9.1%) 9 610 (7.3%)	< 0.0001
**Race**				
White Black Hispanic Asian Other Unknown	416 276 (100%) 45 118 (100%) 4 930 (100%) 5 612 (100%) 9 762 (100%) 14 167 (100%)	381 823 (91.7%) 42 041 (93.2%) 4 573 (92.8%) 5 130 (91.4%) 8 945 (91.6%) 13 060 (92.2%)	34 453 (8.3%) 3 077 (6.8%) 357 (7.2%) 482 (8.6%) 817 (8.4%) 1 107 (7.8%)	< 0.0001
**US Region**				
South West Midwest Northeast	180 800 (100%) 102 364 (100%) 104 985 (100%) 107 716 (100%)	166 529 (92.1%) 93 074 (90.9%) 96 881 (92.3%) 99 088 (92.0%)	14 271 (7.9%) 9 290 (9.1%) 8 104 (7.7%) 8 628 (8.0%)	< 0.0001
**Dual eligibility for Medicaid**				
Yes No	34 267 (100%) 461 598 (100%)	31 687 (92.5%) 423 885 (91.8%)	2 580 (7.5%) 37 713 (8.2%)	< 0.0001
**Disability as the original reason for Medicare entitlement**				
Yes No	43 310 (100%) 452 555 (100%)	39 899 (92.1%) 415 673 (91.9%)	3 411 (7.9%) 36 882 (8.2%)	0.046
**Year of Prostate Cancer Diagnosis**				
2019 2020 2021	212 465 (100%) 144 511 (100%) 138 889 (100%)	193 209 (90.9%) 132 134 (91.4%) 130 229 (93.8%)	19 256 (9.1%) 12 377 (8.6%) 8 660 (6.2%)	< 0.0001
**Number of Chronic Conditions**				
0 1 2 ≥ 3	21 291 (100%) 26 949 (100%) 44 442 (100%) 403 183 (100%)	20 095 (94.4%) 25 292 (93.9%) 41 665 (93.8%) 368 520 (91.4%)	1 196 (5.6%) 1 657 (6.2%) 2 777 (6.3%) 34 663 (8.6%)	< 0.0001

Baseline Characteristics of patients with and without Positron Emission Tomography (PET) scan after the diagnosis of prostate cancer; Percentages are given in row percentages, chi-square test was used to test significance

The radiopharmaceutical used was reported in 79.8% of all PET scans performed. 18 F-FDG and 18 F-fluciclovine were the most commonly used tracers, accounting for 56.8% and 20.0% of all PET scans, respectively. Detailed information on the tracers used for PET scans is provided in Supplementary Table 2.

The odds of obtaining a PET scan were lower for both urban (Odd Ratio [OR] 0.87, 95% Confidence interval [CI] 0.82–0.93) and rural patients (OR 0.87, 95% CI: 0.82–0.92) compared to metro patients. Table 3 summarizes additional independent patient-level covariates of undergoing PET imaging.

**Table 3:** Multivariable logistic regression to predict PET imaging use

	OR	95%CI	*p*-value
**Residence**			
Metro	Ref.	Ref.	
Urban	**0.87**	**0.82–0.93**	**< 0.0001**
Rural	**0.87**	**0.82–0.92**	**< 0.0001**
Unknown*	0.88	0.77–1.02	0.1
**Age Group**			
66–70	Ref.	Ref.	
71–75	**1.05**	**1.02–1.08**	**0.003**
76–80	**1.10**	**1.07–1.14**	**< 0.0001**
> 81	**0.86**	**0.83–0.89**	**< 0.0001**
**Race**			
White	Ref.	Ref.	
Black	**0.82**	**0.78–0.86**	**< 0.0001**
Hispanic	**0.87**	**0.77–0.99**	**0.03**
Asian	1.00	0.90–1.11	0.9
Other	0.97	0.89–1.06	0.5
Unknown	0.95	0.89–1.01	0.1
**US Region**			
South	Ref.	Ref.	
West	1.17	1.04–1.32	0.01
Midwest	0.99	0.92–1.07	0.9
Northeast	1.01	0.90–1.14	0.8
**Dual eligibility for Medicaid**			
Yes	Ref.	Ref.	
No	1.07	0.996–1.14	0.1
**Disability as the original reason for Medicare entitlement**			
Yes	Ref.	Ref.	0.1
No	1.03	0.99–1.08	0.1
**Year of Prostate Cancer Diagnosis**			
2019	Ref.	Ref.	
2020	**0.94**	**0.90–0.97**	**0.0002**
2021	**0.68**	**0.65–0.72**	**< 0.0001**
**Number of Chronic Conditions**			
0	Ref.	Ref.	
1	1.03	0.95–1.11	0.5
2	1.03	0.96–1.11	0.4
≥ 3	**1.45**	**1.36–1.55**	**< 0.0001**

Hospital referral region-clustered multivariable logistic regression for the receipt of Positron Emission Tomography (PET), Abbreviations: OR = odds ratio, 95% CI = 95% Confidence Interval

We found that the interaction term combining race and metro/urban/rural county type was significantly associated with odds of PET imaging (*p* < 0.0001). After adjusting for all covariates, the estimated adjusted probability of White patients obtaining a PET scan was 8.3% in metro, 7.3% in urban, and 7.4% in rural counties. The estimated adjusted probabilities for Black patients undergoing a PET scan were 7.0% in metro, 6.2% in urban, and 5.0% in rural counties.

While the odds of undergoing PET imaging were lower for both Black and White patients living in rural counties, the magnitude of the effect differed. Compared to White metro patients, White rural patients were 12% less likely to receive PET imaging (OR 0.88, 95% CI: 0.83–0.94, *p* = 0.0002). Black rural patients were 30% less likely (0.70, 95% CI: 0.58–0.84, *p* = 0.0002) to undergo a PET scan compared to Black metro patients.

The effect of Black race was attenuated in urban areas. Compared to metro residents, PET scan odds were lower for White urban patients (OR: 0.87, 95% CI: 0.83–0.93, *p* < 0.0001) but not significantly lower for Black urban patients (OR: 0.88, 95% CI: 0.75–1.04, *p* = 0.1).

For Asian, Hispanic and Other patients, there was no impact of residence observed in the odds of receiving a PET scan. Table 4 shows the interaction term logistic regression model and the estimated adjusted probability for Black and White patients living in metro, urban and rural counties to undergo a PET scan.

**Table 4:** Least square means probabilities and interaction odds ratios for PET utilization in a multivariable logistic regression model

	LS Means Probability			ORs with Interaction term (Residence and Race[fixed])		
**Residence**	**Race**	*Adjusted predicted Probability* (LS mean*100)	Adjusted predicted Probability 95%CI *100	*OR*	*95%CI*	*p-value*
**Metro**	Black	7.0%	6.6-7.4%	Ref	Ref	
**Urban**	Black	6.2%	5.4-7.2%	Vs. Metro 0.88	Vs. Metro 0.75–1.04	0.1
**Rural**	Black	5.0%	4.1-5.9%	Vs. Metro 0.70	Vs. Metro 0.58–0.84	0.0002
**Metro**	White	8.3%	8.0-8.6%	Ref	Ref	
**Urban**	White	7.3%	7.0-7.7%	Vs. Metro 0.87	Vs. Metro 0.83–0.93	< 0.0001
**Rural**	White	7.4%	7.0-7.9%	Vs. Metro 0.88	Vs. Metro 0.83–0.94	0.0002

Least square (LS) means adjusted probabilities and residence-race interaction term odds ratios (OR) for undergoing a Postrone Emission Tomography (PET), based on the multivariable logistic regression model clustered by hospital referral regions (HRRs). The first three rows represent Black patient’s residential areas, and the second three rows represent White patient’s residential areas. LS means adjusted probability represents the probability for Black and White patients to receive a PET scan after adjusting for all covariates in the model. The 95% confidence intervals (CI) for the adjusted probabilities are also provided, with the lower and upper bounds calculated using the mean probability and its standard error. The odds ratios (ORs) reflect the differences in PET scan likelihood between residential areas for Black and White patients, as indicated by the interaction terms

## Discussion

In this retrospective analysis of all US Medicare beneficiaries with prostate cancer diagnosed from 2019 to 2021, we observed that patients in rural counties were significantly less likely to undergo PET imaging compared to patients in metro counties. Further, PET imaging was less common among rural patients across all racial groups. However, the association between rural residence and reduced PET imaging access was pronounced among Black patients. In contrast, the racial difference appears to be attenuated in urban counties. Together, our findings reveal geographic differences in the use of advanced diagnostics for patients with prostate cancer enrolled in Medicare.

Our analysis showed statistically significant differences in the proportional use of PET scans between metro, urban, and rural counties. These findings align with evidence showing rural patients have reduced healthcare access and worse prostate cancer care, including fewer biopsy cores and multidisciplinary consultations [2, 24, 25]. While data on disease stage at diagnosis shows conflicting results, a systematic review suggests rural patients tend to present with more advanced disease [26]. Given the primary use of PET imaging in a limited cohort of higher-risk patients, one could argue that the use of PET scans in rural areas should be even higher than in urban areas.

Yet, the rural–urban gap and regional variability may be particularly pronounced for advanced imaging procedures [27]. A study of MRI imaging for prostate cancer demonstrated that significant rural-urban differences remained constant from 2012 to 2019. Although MRI use increased in both urban and rural locations, rural patients were overall 35% less likely to undergo MRI imaging [28]. Unlike alternatives for optimal treatment planning or counseling, such as telemedicine, advanced imaging (including PET) requires on-site equipment and healthcare resources. Further, PET isotopes are produced in a cyclotron or radionuclide generator and transported to imaging facilities for patient injection. Due to their short half-life, some injections expire within hours, potentially limiting access in rural areas where longer distances complicate logistics. This infrastructure requirement may contribute to disparities in advanced diagnostic access across metro, urban, and rural regions [5]. The regional dependency is possibly reflected in our study’s high variability in PET scan rates, ranging from 2.2 to 20.8% across HRRs, likely reflecting differences in infrastructure and regional practices. Various strategies are currently being explored to enhance access to advanced imaging procedures, with imaging networks assisted by teleradiology emerging as a particularly promising approach [29, 30]. Establishing hub-and-spoke structures may be particularly beneficial for advanced imaging referral in rural communities [31]. In this model, a central facility (hub) provides comprehensive services, while smaller sites (spokes) offer basic diagnostics and could refer patients to the hub [32]. Such networks are already implemented in imaging and treatment, aiming to coordinate care delivery, improve efficiency, and maintain high standards across regions [31, 33]. This model can also incorporate teleradiology to support appropriate referrals and triage. By expanding the availability of subspecialized radiologists, teleradiology can improve not only imaging accessibility but also diagnostic quality, as recently demonstrated in advanced prostate imaging [34–36]. A recent national survey found that 78% of radiologists currently perform teleradiology services, including 46% who provide interpretations for rural areas [37]. Another approach to overcome disparities is the use of mobile imaging units, specialized vehicles equipped with imaging technologies [38, 39]. Following the implementation of mobile mammography units in Canada in 2002, mammography participation rates were higher in regions served only by mobile units compared to regions with both mobile and fixed centers. In regions served exclusively by mobile units, these mobile services accounted for 90.6% of the overall participation rate [40].

Our findings also reveal racial and ethnic differences in the use of PET scans. These disparities in prostate cancer care occur early and persist throughout the disease course, resulting in worse outcomes [1]. Black and Hispanic patients are more likely to be diagnosed with advanced-stage prostate cancer and experience imaging and treatment delays [41–43]. Given their higher risk profiles, these patients should undergo PET staging more frequently, particularly since addressing early racial disparities could improve overall outcomes [1]. Conversely, evidence suggests that Black and Hispanic patients have lower rates of diagnostic imaging procedures, which is consistent with our findings. For instance, Black patients were shown to be 43.9% less likely to receive prostate MRI after an elevated Prostate Specific Antigen (PSA) test [44]. Geographic residence added an important dimension to observed racial differences. While the residence effect was consistent across Black and White beneficiaries, Black beneficiaries in rural counties had the lowest estimated probability of obtaining a PET scan. These structural patterns are also seen in prostate MRI among diagnosed patients, where Black patients were 38% less likely to receive MRI, with 81% of racial disparities mediated by sociodemographic factors including geography (24%), residential segregation (19%), and census-based poverty (19%) [45]. Patients in more urbanized areas could benefit from better healthcare infrastructure, including access to academic centers, imaging facilities, and social support services [3, 46].

There are several important considerations that must be taken into account when interpreting our results. First, staging procedures by whole-body imaging are indicated only in selected patients with newly diagnosed prostate cancer. International guidelines do not recommend staging, whether by conventional imaging or PET, in patients with low-risk and favorable intermediate-risk disease [47, 48]. In a population-based study, approximately 75% of patients with prostate cancer initially presented with low or intermediate risk disease [49]. Our cohort included all Medicare beneficiaries with newly diagnosed prostate cancer, though whole-body imaging is needed for only a subset of patients. Therefore, our results could reflect selective staging in intermediate-risk disease. However, rural-urban differences may be even greater when considering only a limited cohort requiring whole-body imaging.

Second, PET imaging was not a standard diagnostic tool for prostate cancer, although its role is evolving due to its diagnostic capability [8]. Furthermore, several tracers have been approved in recent years, and their use in our study may reflect their regulatory approval status. 18 F-fluorodeoxyglucose, 11 C-choline, and 18 F-fluciclovine were available throughout the study period, while 68Ga-PSMA-11 and 18 F-piflufolastat received U.S. Food and Drug Administration (FDA) approval in December 2020 and May 2021, respectively. Our findings indicate that non-PSMA PET imaging was commonly used during the study period, with most scans performed around the time of diagnosis. This may reflect a willingness among clinicians to adopt advanced diagnostic tools, despite limited tracer availability or evolving consensus on optimal clinical use. These observations underscore the evolving role of PET imaging and the urgent need to address access disparities as more effective radiopharmaceuticals, particularly PSMA-based tracers, become central to clinical practice.

We observed fewer prostate cancer diagnoses in 2020 and 2021 compared to 2019. Our study included all PET scans performed within the study period without time-based censoring, accounting for those affected by COVID-19 pandemic delays. The pandemic significantly impacted diagnostic imaging, with non-urgent imaging procedures experiencing worldwide reductions and pre-existing diagnostic radiology disparities being exacerbated [50–52]. One single academic center study reported PET-CT imaging was particularly affected with a 62.5% reduction at the nadir [53]. Further, consistent with our data, a meta-analysis reported cancer diagnoses globally declined by 27% during the pandemic, with prostate cancer specifically declining by 26.2% and more pronounced reductions in Non-Hispanic Black patients [54]. The lower PET utilization in patients diagnosed in 2020 and 2021 in our study likely reflects both pandemic-related disruptions and limited follow-up time in later diagnoses years.

A limitation in interpreting our results is the absence of clinicopathologic data in Medicare claims, preventing assessment of whether PET utilization reflects appropriate clinical indications or broader access disparities. While Surveillance, Epidemiology, and End Results Program (SEER)-Medicare would provide essential tumor characteristics and risk stratification, this dataset was unsuitable for our geographic analysis due to its restriction to 18 registries, exclusion of large rural states, and documented differences in travel distances to healthcare facilities between SEER and non-SEER populations [55, 56]. These travel time disparities are particularly pronounced in rural areas, making SEER-Medicare inadequate for examining the rural-urban differences that were central to our study objectives [57]. The cohort included only Medicare beneficiaries aged 65 and older, limiting generalizability to younger or non-Medicare populations. As universally insured individuals, the true disparities in PET use may be greater than observed. We used Medicare’s original race coding, which may lack granularity, potentially affecting numerical outcomes. Further, it is possible that some patients underwent PET imaging for reasons unrelated to prostate cancer diagnosis or staging.

However, a strength of this study is the large, real-world database of all claims for patients with newly diagnosed prostate cancer in the Medicare population between 2019 and 2021. All newly diagnosed prostate cancer patients were included to provide the most comprehensive picture of early PET use.

## Conclusion

This study highlights geographic disparities in the use of PET imaging among patients with prostate cancer. Rural and urban residents are less likely to undergo PET scans compared to their metro counterparts. In addition, Black patients experienced a more pronounced effect of rurality on lower odds of undergoing PET scans. While this study aimed to address disparities in the early adoption of PET imaging for prostate cancer, the findings underscore the need for further health services research. PET imaging offers advanced diagnostic capabilities, enabling accurate staging and determining eligibility for life-prolonging, FDA-approved radionuclide therapy. The observed disparities highlight the importance of targeted initiatives to improve access to advanced diagnostics, especially addressing geographic and racial inequities. Future work should explore patient- and provider-level barriers and evaluate implementation strategies to reduce rural–urban differences in advanced prostate cancer care.

## Electronic supplementary material

Below is the link to the electronic supplementary material.

Supplementary Material 1

## Data Availability

For this study, we accessed Medicare claims data through a licensed agreement with the Centers for Medicare & Medicaid Services (CMS). In accordance with the standard CMS data use agreement, we are not permitted to share the data directly.

## Abbreviations

11 C-Choline: Carbon-11 choline
18 F-FDG: Fluorine-18 fluorodeoxyglucose
18 F-fluciclovine: Fluorine-18 fluciclovine
18 F-piflufolastat: Fluorine-18 piflufolastat
68Ga-PSMA-11: Gallium-68 prostate-specific membrane antigen-11
CI: Confidence interval
FDA: U.S. food and drug administration
HRR: Hospital referral region
ICD-10: International classification of diseases, 10th revision
IQR: Interquartile range
MRI: Magnetic resonance imaging
OR: Odds ratio
PET: Positron emission tomography
PSA: Prostate-specific antigen
PSMA: Prostate-specific membrane antigen
SEER: Surveillance, epidemiology, and end results program
