# Supplementary material for: Geographic variability in contemporary utilization of PET imaging for prostate cancer: a medicare claims cohort study
Source: Cancer Imaging. 2025 Jul 4;25:86. doi: 10.1186/s40644-025-00898-6 (PMC12231898; doi:10.1186/s40644-025-00898-6)
Supplement: Supplementary file 1 — Supplementary Material 1 [file 40644_2025_898_MOESM1_ESM.docx]

**Supplementary Appendix**

This study examines geographic disparities in PET imaging utilization among Medicare beneficiaries with prostate cancer (2019-2021). The supplementary appendix includes a national heatmap of PET rates, patient selection flowchart, temporal scan distribution, PET tracer distribution, coding definitions, and rural-urban classification criteria.

Supplementary Figure 1.


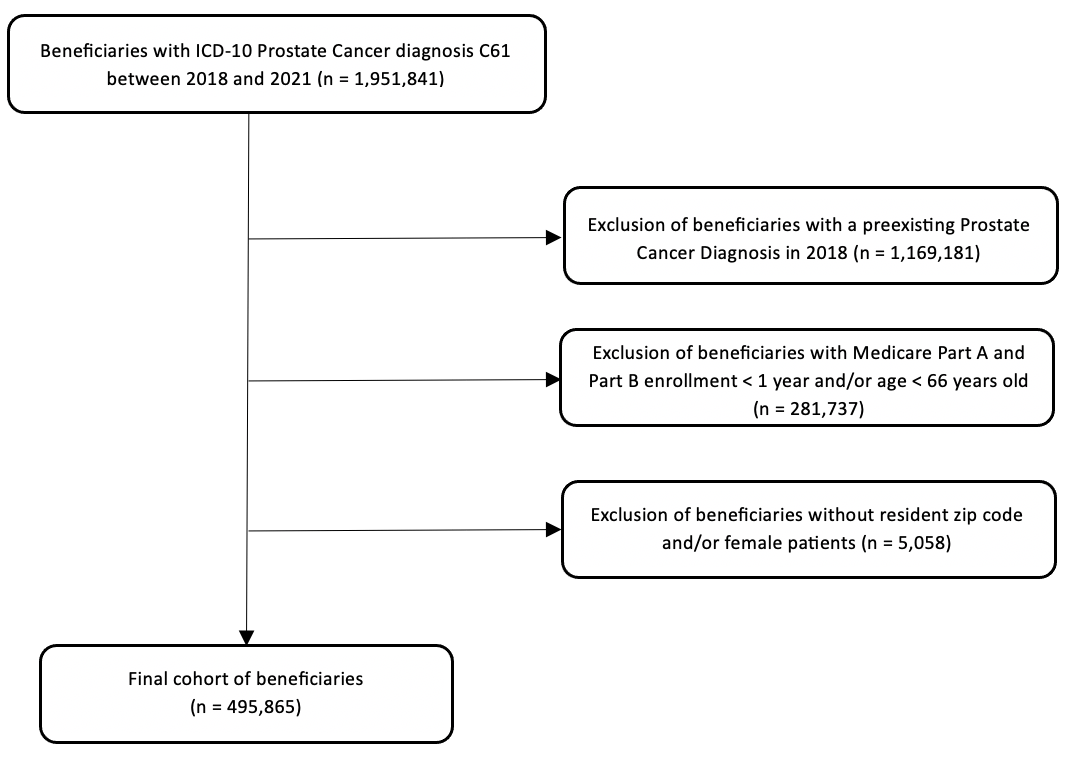


Supplementary Figure 1, Materials and Methods, Flow chart of the study population selection process of Medicare beneficiaries with and without PET scan included in our analysis.

Supplementary Figure 2


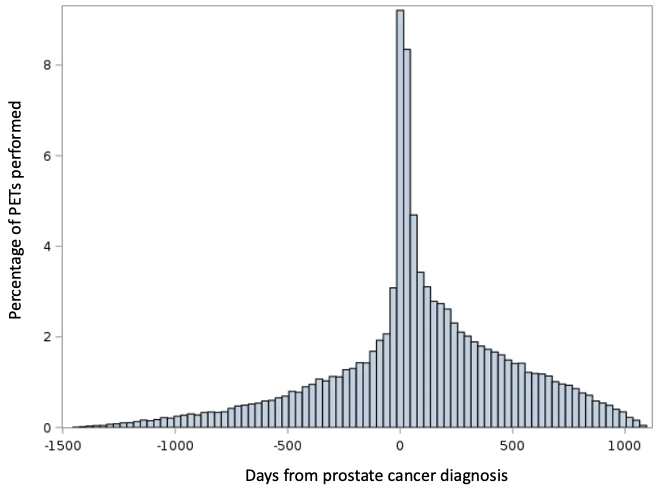


Supplementary Figure 2, Temporal pattern validation analysis. Distribution of Positron Emission Tomography (PET) Scans Relative to Prostate cancer Diagnosis Date.

| **Supplementary Table 1. Codes for PET claims** | |
| --- | --- |
|  | CPT/HCPCS Codes |
| CPT/HCPCS Codes included to identify patients with PET imaging, at least one code or a combination of these codes was required to identify patients. | 78801, 78802, 78803, 78804, 78811, 78812, 78813, 78814, 78815, 78816, 78830,78831, 78832, 78835, 78999, A9515, A9552, A9588, A9593, A9594, A9595, G0235, |

Supplementary Table 1, Materials and Methods, Code set for Positron Emission Tomography (PET) identification, Current Procedural Terminology (CPT)/Healthcare Common Procedure Coding System (HCPCS)

| Supplementary Table 2. Residence Classification | | | |
| --- | --- | --- | --- |
| Study Category | Rural Urban Continuum Code 2023 | Description | Original Classification |
| Metro | 1 | Counties in metropolitan areas ≥1 million population | Metro |
| Metro | 2 | Counties in metropolitan areas 250,000-1 million population | Metro |
| Metro | 3 | Counties in metropolitan areas <250,000 population | Metro |
| Urban | 4 | Nonmetro counties ≥20,000 population, adjacent to metro area | Nonmetro |
| Urban | 5 | Nonmetro counties ≥20,000 population, not adjacent to metro area | Nonmetro |
| Urban | 6 | Nonmetro counties 5,000-20,000 population, adjacent to metro area | Nonmetro |
| Rural | 7 | Nonmetro counties 5,000-20,000 population, not adjacent to metro area | Nonmetro |
| Rural | 8 | Nonmetro counties <5,000 population, adjacent to metro area | Nonmetro |
| Rural | 9 | Nonmetro counties <5,000 population, not adjacent to metro area | Nonmetro |

Supplementary Table 2, Materials and Methods, Rural-Urban Continuum Codes by the US Department of Agriculture^15^ and the used definition of metro, urban and rural areas

| Supplementary Table 3. Distribution of PET Tracers | | |
| --- | --- | --- |
|  | Overall patients number (n) | % of all PET scans performed |
| Overall PET scans performed | **40,293** | **100%** |
| Tracer indicated | **32,135** | **79.8%** |
| 18F-Fluorodeoxyglucose | 22,872 | 56.8% |
| 18F-Fluciclovine | 8,043 | 20.0% |
| C11-Choline | 231 | 0.6% |
| 68Ga-PSMA-11 | 152 | 0.4% |
| 18F-Piflufolastat | 0 | 0.0% |
| Combination of tracers indicated | 837 | 2.1% |
| Tracer not indicated | **8,158** | **20.2%** |

Supplementary Table 3, Distribution of Positron Emission Tomography (PET) tracers indicated in identified patients undergoing a PET scan, Abbrevations: PSMA prostate specific membrane antigen
